# The genome sequence of the Oak Nycteoline moth, *Nycteola revayana *(Scopoli, 1772)

**DOI:** 10.12688/wellcomeopenres.21567.1

**Published:** 2024-05-15

**Authors:** Douglas Boyes, Gavin R. Broad, Peter W. H. Holland, University of Oxford and Wytham Woods Genome Acquisition Lab, Natural History Museum Genome Acquisition Lab, Darwin Tree of Life Barcoding collective, Wellcome Sanger Institute Tree of Life Management, Samples and Laboratory team, Wellcome Sanger Institute Tree of Life Core Informatics team, Tree of Life Core Informatics collective, Darwin Tree of Life Consortium

**Affiliations:** 1 UK Centre for Ecology & Hydrology, Wallingford, England, UK; 2 Natural History Museum, London, England, UK; 3 University of Oxford, Oxford, England, UK

**Keywords:** Nycteola revayana, Oak Nycteoline moth, genome sequence, chromosomal, Lepidoptera

## Abstract

We present a genome assembly from an individual male *Nycteola revayana* (the Oak Nycteoline moth; Arthropoda; Insecta; Lepidoptera; Nolidae). The genome sequence is 621.0 megabases in span. Most of the assembly is scaffolded into 26 chromosomal pseudomolecules, including the Z sex chromosome. The mitochondrial genome has also been assembled and is 15.25 kilobases in length. Gene annotation of this assembly on Ensembl identified 19,235 protein-coding genes.

## Species taxonomy

Eukaryota; Opisthokonta; Metazoa; Eumetazoa; Bilateria; Protostomia; Ecdysozoa; Panarthropoda; Arthropoda; Mandibulata; Pancrustacea; Hexapoda; Insecta; Dicondylia; Pterygota; Neoptera; Endopterygota; Amphiesmenoptera; Lepidoptera; Glossata; Neolepidoptera; Heteroneura; Ditrysia; Obtectomera; Noctuoidea; Nolidae; Chloephorinae; *Nycteola*; *Nycteola revayana* (Scopoli, 1772) (NCBI:txid988004).

## Background

The Oak Nycteoline *Nycteola revayana* is a small moth in the family Nolidae, superfamily Noctuoidea, found widely across western, central and northern Europe, with scattered records from Russia and north Africa ( GBIF Secretariat, 2023). In Britain and Ireland, the moth is recorded most frequently in southern and central England and Wales but rarely in large numbers; it has a scattered distribution across Scotland and is scarce in Northern Ireland and Ireland ( GBIF Secretariat, 2023; NBN Atlas Partnership, 2023; Randle *et al.*, 2019). An unusual feature of this species is the extensive variation in ground colour of the wings and in wing pattern. For example, moths may be ashy grey with indistinct markings, bronze-brown with pronounced black streaks running from the wing base, pale grey with ‘domino-like’ spots, or crossed by bands of brown, black and grey. In 1919, W.G. Sheldon combined previous work with examination of additional specimens to divide the species into 30 named ‘varieties’ ( Sheldon, 1919). However, since intermediate forms exist, these may be better considered simply as combinations of variants in several independent wing pattern elements, rather than distinct morphs with co-inherited features.

The larvae of *N. revayana* feed on pedunculate oak *Quercus robur* and sessile oak *Q. petraea*, and the moth is found in deciduous woodlands or in fields, parks and hedgerows where oaks are present. In Britain, the species is thought to be primarily bivoltine: one generation is on the wing in autumn and spring, overwintering as an adult, with the eggs laid by these moths developing into a summer generation with more individuals. However, this pattern may be overlain by some moths having a univoltine life history, and supplemented by migrant moths from mainland Europe ( DorsetMoths, 2023; Randle *et al.*, 2019).

A genome sequence for *N. revayana* will facilitate studies into adaptations to oak feeding and the genetic basis of wing pattern variation, and will contribute to the growing set of genomic resources for Lepidoptera.

## Genome sequence report

**Figure 1.**
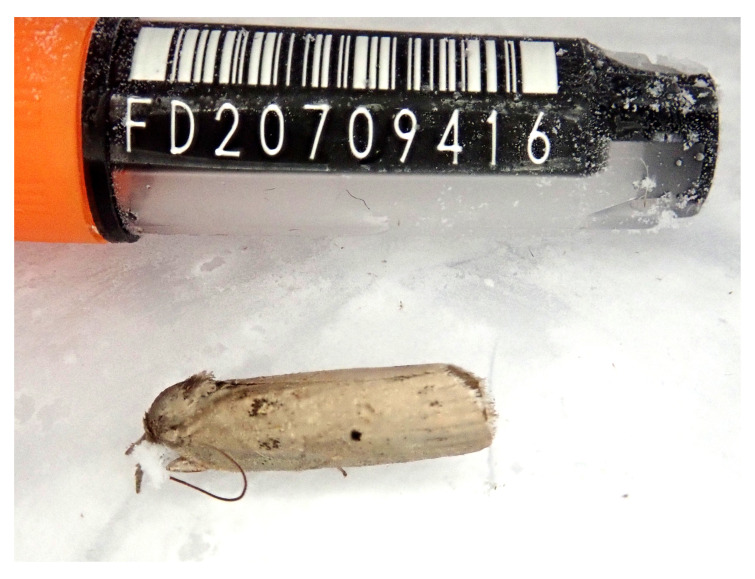
Photograph of the *Nycteola revayana* (ilNycReva1) specimen used for genome sequencing.

The genome was sequenced from a male *Nycteola revayana* ( Figure 1) collected from Wytham Woods, Oxfordshire, UK (51.77, –1.34). A total of 33-fold coverage in Pacific Biosciences single-molecule HiFi long reads was generated. Primary assembly contigs were scaffolded with chromosome conformation Hi-C data. Manual assembly curation corrected 8 missing joins or mis-joins and removed 2 haplotypic duplications, reducing the scaffold number by 4.26%.

**Figure 2.**
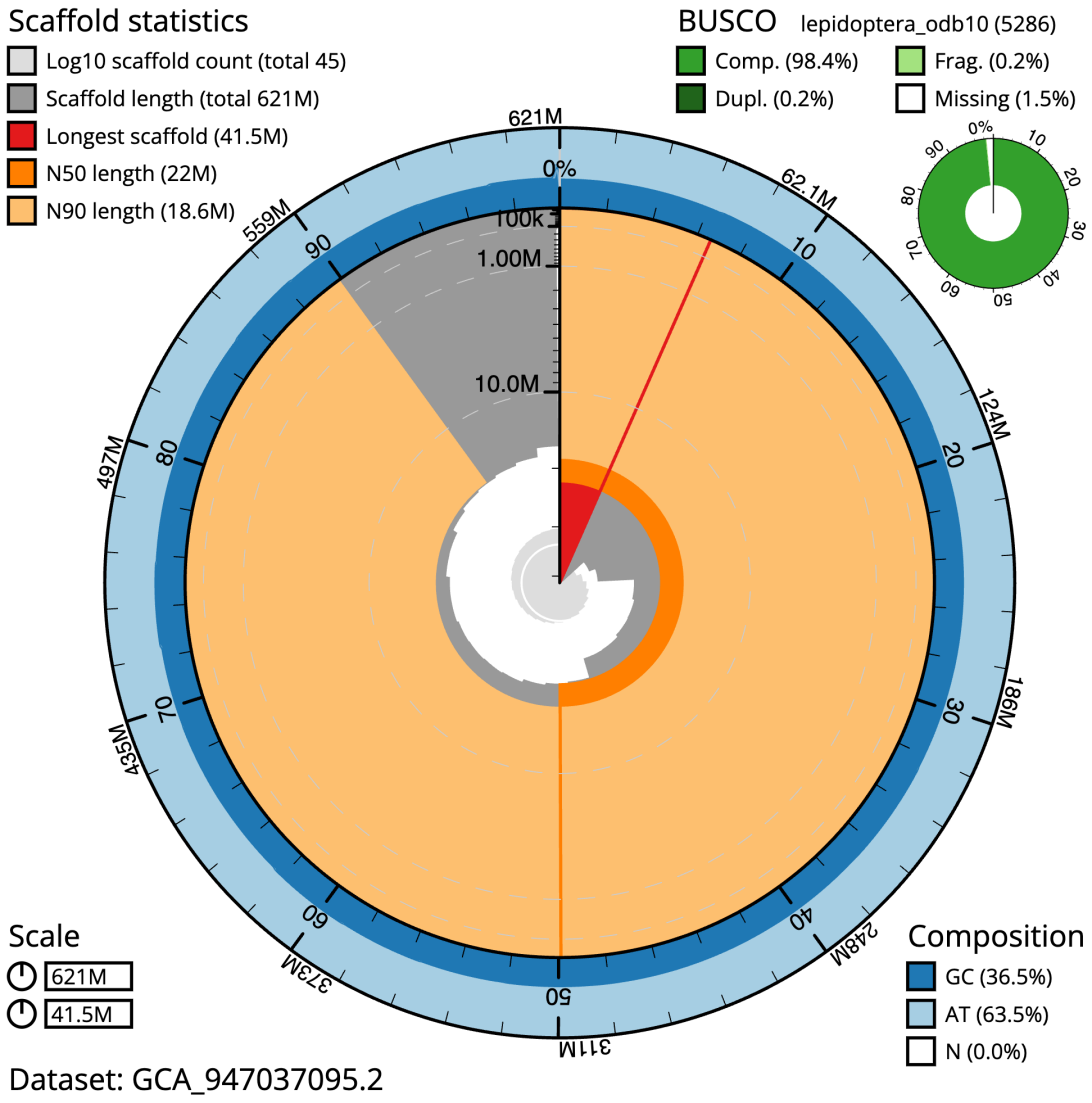
Genome assembly of *Nycteola revayana*, ilNycReva1.2: metrics. The BlobToolKit snail plot shows N50 metrics and BUSCO gene completeness. The main plot is divided into 1,000 size-ordered bins around the circumference with each bin representing 0.1% of the 621,039,493 bp assembly. The distribution of scaffold lengths is shown in dark grey with the plot radius scaled to the longest scaffold present in the assembly (41,453,523 bp, shown in red). Orange and pale-orange arcs show the N50 and N90 scaffold lengths (21,960,024 and 18,563,743 bp), respectively. The pale grey spiral shows the cumulative scaffold count on a log scale with white scale lines showing successive orders of magnitude. The blue and pale-blue area around the outside of the plot shows the distribution of GC, AT and N percentages in the same bins as the inner plot. A summary of complete, fragmented, duplicated and missing BUSCO genes in the lepidoptera_odb10 set is shown in the top right. An interactive version of this figure is available at https://blobtoolkit.genomehubs.org/view/Nycteola_revayana/dataset/GCA_947037095.2/snail.

**Figure 3.**
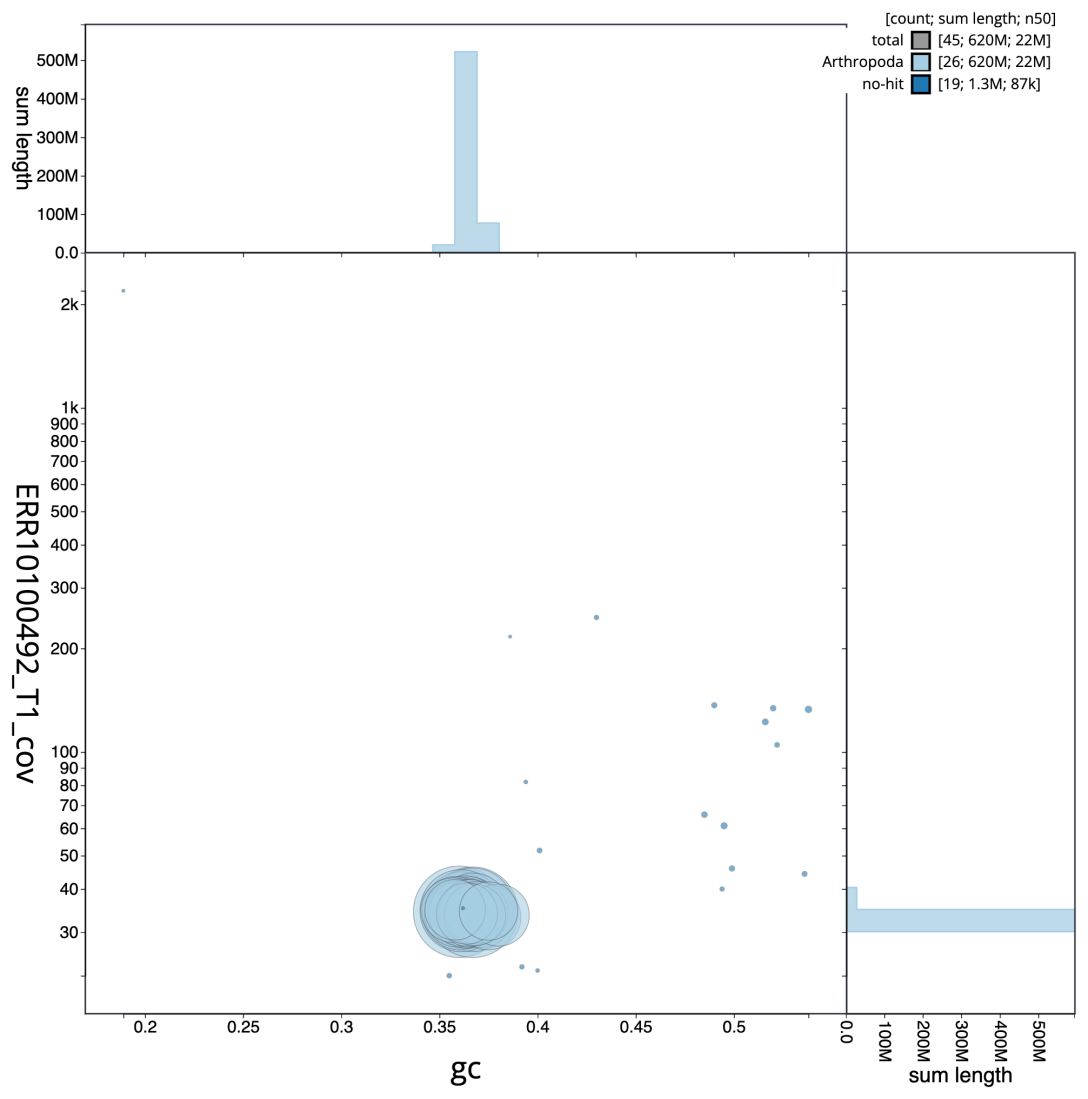
Genome assembly of *Nycteola revayana*, ilNycReva1.2: BlobToolKit GC-coverage plot. Sequences are coloured by phylum. Circles are sized in proportion to sequence length. Histograms show the distribution of sequence length sum along each axis. An interactive version of this figure is available at https://blobtoolkit.genomehubs.org/view/Nycteola_revayana/dataset/GCA_947037095.2/blob.

**Figure 4.**
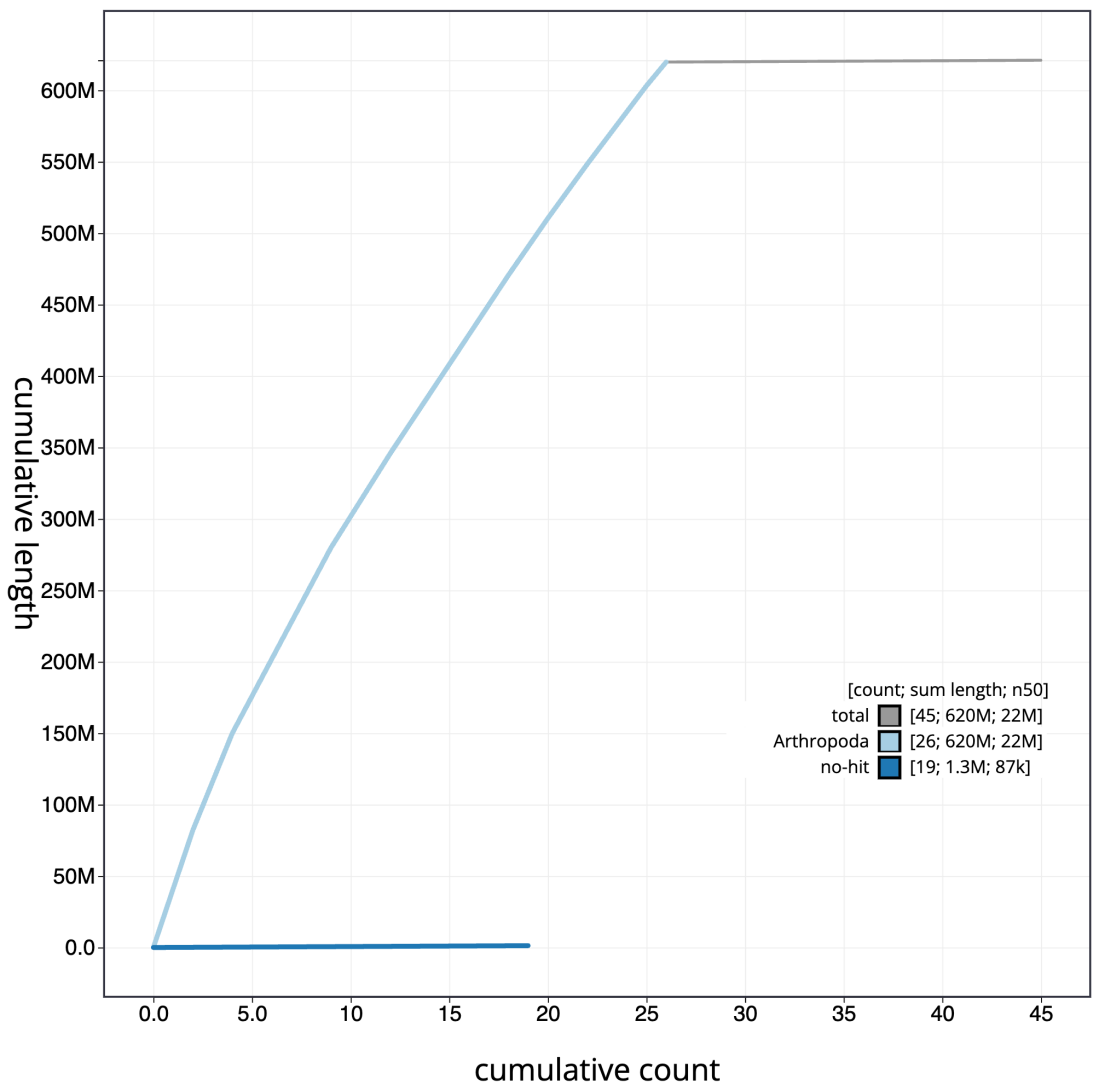
Genome assembly of *Nycteola revayana*, ilNycReva1.2: BlobToolKit cumulative sequence plot. The grey line shows cumulative length for all sequences. Coloured lines show cumulative lengths of sequences assigned to each phylum using the buscogenes taxrule. An interactive version of this figure is available at https://blobtoolkit.genomehubs.org/view/Nycteola_revayana/dataset/GCA_947037095.2/cumulative.

**Figure 5.**
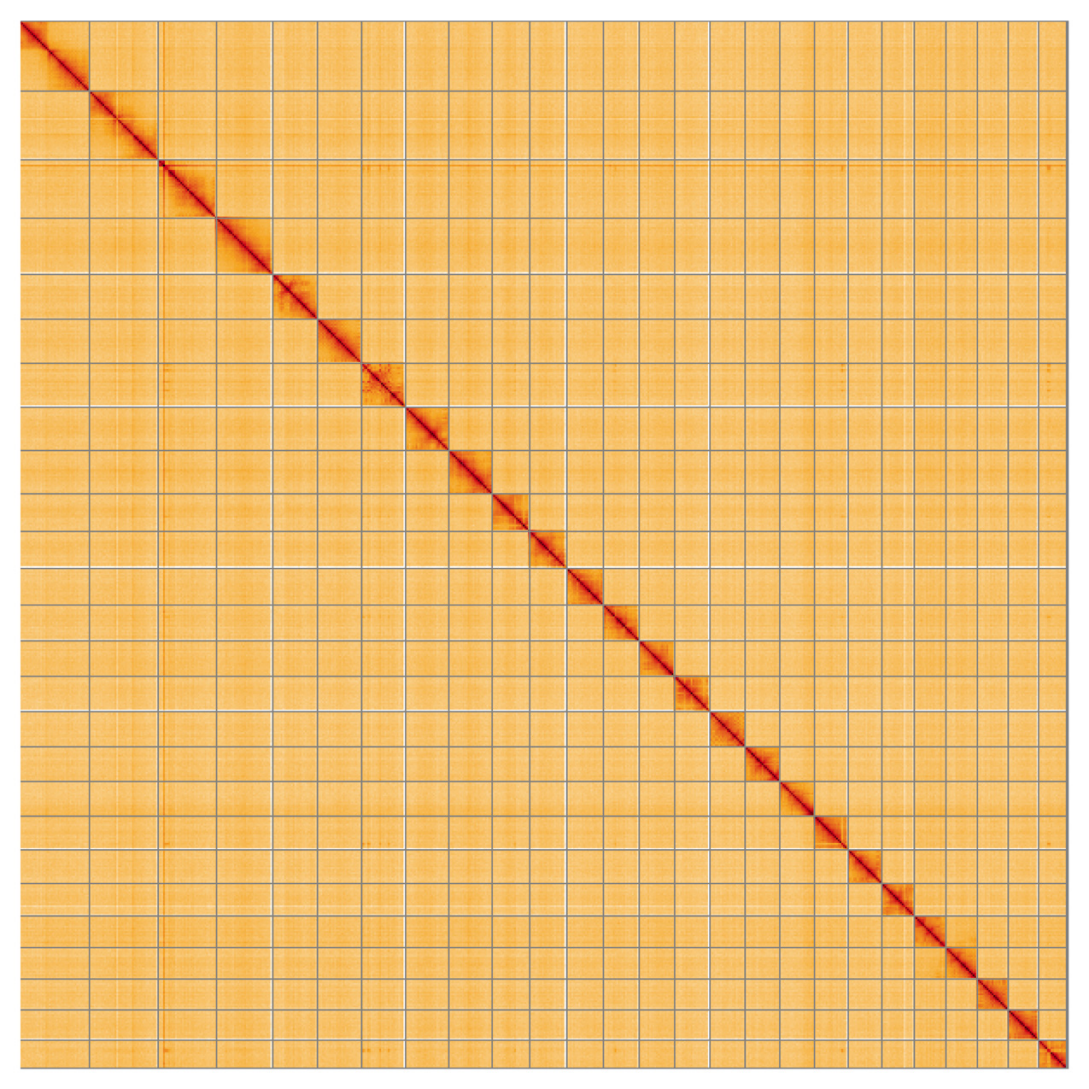
Genome assembly of *Nycteola revayana*, ilNycReva1.2: Hi-C contact map of the ilNycReva1.2 assembly, visualised using HiGlass. Chromosomes are shown in order of size from left to right and top to bottom. An interactive version of this figure may be viewed at https://genome-note-higlass.tol.sanger.ac.uk/l/?d=EP63k2ELTVC8tQ8OwXI0mg.

The final assembly has a total length of 621.0 Mb in 44 sequence scaffolds with a scaffold N50 of 22.0 Mb ( Table 1). The snail plot in Figure 2 provides a summary of the assembly statistics, while the distribution of assembly scaffolds on GC proportion and coverage is shown in Figure 3. The cumulative assembly plot in Figure 4 shows curves for subsets of scaffolds assigned to different phyla. Most (99.79%) of the assembly sequence was assigned to 26 chromosomal-level scaffolds, representing 24 autosomes and the Z sex chromosome. Chromosome-scale scaffolds confirmed by the Hi-C data are named in order of size ( Figure 5; Table 2). While not fully phased, the assembly deposited is of one haplotype. Contigs corresponding to the second haplotype have also been deposited. The mitochondrial genome was also assembled and can be found as a contig within the multifasta file of the genome submission.

**Table 1.** Genome data for *Nycteola revayana*, ilNycReva1.2.

Project accession data		
Assembly identifier	ilNycReva1.2	
Species	*Nycteola revayana*	
Specimen	ilNycReva1	
NCBI taxonomy ID	988004	
BioProject	PRJEB55450	
BioSample ID	SAMEA10107012	
Isolate information	ilNycReva1, male: whole organism (PacBio DNA sequencing) ilNycReva2, male: whole organism (Illumina Hi-C sequencing) ilNycReva3: abdomen (RNA sequencing)	
Assembly metrics *		*Benchmark*
Consensus quality (QV)	66.3	*≥ 50*
*k*-mer completeness	100.0%	*≥ 95%*
BUSCO **	C:98.4%[S:98.2%,D:0.2%], F:0.2%,M:1.4%,n:5,286	*C ≥ 95%*
Percentage of assembly mapped to chromosomes	99.79%	*≥ 95%*
Sex chromosomes	Z	*localised homologous pairs*
Organelles	Mitochondrial genome: 15.25 kb	*complete single alleles*
Raw data accessions		
PacificBiosciences Sequel IIe	ERR10100492	
Hi-C Illumina	ERR10107966	
PolyA RNA-Seq Illumina	ERR12708729	
Genome assembly		
Assembly accession	GCA_947037095.2	
*Accession of alternate haplotype*	GCA_947038005.1	
Span (Mb)	621.0	
Number of contigs	110	
Contig N50 length (Mb)	11.3	
Number of scaffolds	44	
Scaffold N50 length (Mb)	22.0	
Longest scaffold (Mb)	41.45	
Genome annotation		
Number of protein-coding genes	19,235	
Number of gene transcripts	19,452	

* Assembly metric benchmarks are adapted from column VGP-2020 of “Table 1: Proposed standards and metrics for defining genome assembly quality” from Rhie *et al.* (2021). ** BUSCO scores based on the lepidoptera_odb10 BUSCO set using version v5.4.3. C = complete [S = single copy, D = duplicated], F = fragmented, M = missing, n = number of orthologues in comparison. A full set of BUSCO scores is available at https://blobtoolkit.genomehubs.org/view/Nycteola_revayana/dataset/GCA_947037095.2/busco.

**Table 2.** Chromosomal pseudomolecules in the genome assembly of *Nycteola revayana*, ilNycReva1.

INSDC accession	Chromosome	Length (Mb)	GC%
OX344838.1	1	41.45	36.0
OX344839.1	2	40.64	36.5
OX344840.2	3	34.57	36.5
OX344841.2	4	26.46	36.0
OX344843.2	5	26.1	36.0
OX344844.2	6	26.03	36.5
OX344845.2	7	25.63	36.5
OX344846.2	8	25.58	36.5
OX344847.2	9	22.26	36.5
OX344848.2	10	21.96	36.0
OX344849.2	11	21.64	36.0
OX344850.2	12	21.17	36.5
OX344851.2	13	21.02	37.0
OX344852.2	14	20.89	36.0
OX344853.2	15	20.8	36.0
OX344854.2	16	20.55	35.5
OX344855.2	17	20.54	37.5
OX344856.2	18	19.94	36.5
OX344857.2	19	19.9	36.5
OX344858.2	20	19.2	37.0
OX344859.2	21	18.77	38.0
OX344860.2	22	18.56	37.0
OX344861.2	23	18.29	36.5
OX344862.2	24	17.91	36.0
OY855748.1	25	16.62	37.5
OX344842.1	Z	33.29	36.0
OX344863.1	MT	0.02	19.5

The estimated Quality Value (QV) of the final assembly is 66.3 with *k*-mer completeness of 100.0%, and the assembly has a BUSCO v completeness of 98.4% (single = 98.2%, duplicated = 0.2%), using the lepidoptera_odb10 reference set ( *n* = 5,286).

Metadata for specimens, BOLD barcode results, spectra estimates, sequencing runs, contaminants and pre-curation assembly statistics are given at https://links.tol.sanger.ac.uk/species/988004.

## Genome annotation report

The *Nycteola revayana* genome assembly (GCA_947037095.1) was annotated at the European Bioinformatics Institute (EBI) on Ensembl Rapid Release. The resulting annotation includes 19,453 transcribed mRNAs from 19,235 protein-coding genes ( Table 1; https://rapid.ensembl.org/Nycteola_revayana_GCA_947037095.1/Info/Index).

## Methods

### Sample acquisition and nucleic acid extraction

Two *Nycteola revayana* (ilNycReva1 and ilNycReva2) were collected from Wytham Woods, Oxfordshire (biological vice-county: Berkshire), UK (latitude 51.77, longitude –1.34) on 2021-03-31 by Douglas Boyes (University of Oxford), using a light trap. The specimens were identified by the collector and then snap-frozen on dry ice. Individual ilNycReva1 (specimen Ox001089) was used for genome sequencing, while ilNycReva2 (specimen Ox001090) was used for Hi-C scaffolding.

The specimen used for RNA sequencing (specimen ID NHMUK010636607, ToLID ilNycReva3) was collected from Tonbridge, Kent, UK (latitude 51.19, longitude 0.29) on 2022-03-10 by Gavin Broad (Natural History Museum), using actinic light.

The workflow for high molecular weight (HMW) DNA extraction at the Wellcome Sanger Institute (WSI) Tree of Life Core Laboratory includes a sequence of core procedures: sample preparation; sample homogenisation, DNA extraction, fragmentation, and clean-up. In sample preparation, the ilNycReva1 sample was weighed and dissected on dry ice ( Jay *et al.*, 2023). Tissue from the whole organism was homogenised using a PowerMasher II tissue disruptor ( Denton *et al.*, 2023a). HMW DNA was extracted using the Automated MagAttract v1 protocol ( Sheerin *et al.*, 2023). DNA was sheared into an average fragment size of 12–20 kb in a Megaruptor 3 system with speed setting 30 ( Todorovic *et al.*, 2023). Sheared DNA was purified by solid-phase reversible immobilisation ( Strickland *et al.*, 2023): in brief, the method employs a 1.8X ratio of AMPure PB beads to sample to eliminate shorter fragments and concentrate the DNA. The concentration of the sheared and purified DNA was assessed using a Nanodrop spectrophotometer and Qubit Fluorometer and Qubit dsDNA High Sensitivity Assay kit. Fragment size distribution was evaluated by running the sample on the FemtoPulse system.

RNA was extracted from abdomen tissue of ilNycReva3 in the Tree of Life Laboratory at the WSI using the RNA Extraction: Automated MagMax™ *mir*Vana protocol ( do Amaral *et al.*, 2023). The RNA concentration was assessed using a Nanodrop spectrophotometer and a Qubit Fluorometer using the Qubit RNA Broad-Range Assay kit. Analysis of the integrity of the RNA was done using the Agilent RNA 6000 Pico Kit and Eukaryotic Total RNA assay.

Protocols developed by the WSI Tree of Life laboratory are publicly available on protocols.io ( Denton *et al.*, 2023b).

### Sequencing

Pacific Biosciences HiFi circular consensus DNA sequencing libraries were constructed according to the manufacturers’ instructions. Poly(A) RNA-Seq libraries were constructed using the NEB Ultra II RNA Library Prep kit. DNA and RNA sequencing was performed by the Scientific Operations core at the WSI on Pacific Biosciences Sequel IIe (HiFi) and Illumina NovaSeq 6000 (RNA-Seq) instruments. Hi-C data were also generated from $HIC_TISSUE tissue of ilNycReva2 using the Arima2 kit and sequenced on the Illumina NovaSeq 6000 instrument.

### Genome assembly and curation

Assembly was carried out with Hifiasm ( Cheng *et al.*, 2021) and haplotypic duplication was identified and removed with purge_dups ( Guan *et al.*, 2020). The assembly was then scaffolded with Hi-C data ( Rao *et al.*, 2014) using YaHS ( Zhou *et al.*, 2023). The assembly was checked for contamination and corrected using the gEVAL system ( Chow *et al.*, 2016) as described previously ( Howe *et al.*, 2021). Manual curation was performed using gEVAL, HiGlass ( Kerpedjiev *et al.*, 2018) and PretextView ( Harry, 2022). The mitochondrial genome was assembled using MitoHiFi ( Uliano-Silva *et al.*, 2023), which runs MitoFinder ( Allio *et al.*, 2020) or MITOS ( Bernt *et al.*, 2013) and uses these annotations to select the final mitochondrial contig and to ensure the general quality of the sequence.

### Final assembly evaluation

The final assembly was post-processed and evaluated with the three Nextflow ( Di Tommaso *et al.*, 2017) DSL2 pipelines “sanger-tol/readmapping” ( Surana *et al.*, 2023a), “sanger-tol/genomenote” ( Surana *et al.*, 2023b), and “sanger-tol/blobtoolkit” ( Muffato *et al.*, 2024). The pipeline sanger-tol/readmapping aligns the Hi-C reads with bwa-mem2 ( Vasimuddin *et al.*, 2019) and combines the alignment files with SAMtools ( Danecek *et al.*, 2021). The sanger-tol/genomenote pipeline transforms the Hi-C alignments into a contact map with BEDTools ( Quinlan & Hall, 2010) and the Cooler tool suite ( Abdennur & Mirny, 2020), which is then visualised with HiGlass ( Kerpedjiev *et al.*, 2018). It also provides statistics about the assembly with the NCBI datasets ( Sayers *et al.*, 2024) report, computes *k*-mer completeness and QV consensus quality values with FastK and MerquryFK, and a completeness assessment with BUSCO ( Manni *et al.*, 2021).

The sanger-tol/blobtoolkit pipeline is a Nextflow port of the previous Snakemake Blobtoolkit pipeline ( Challis *et al.*, 2020). It aligns the PacBio reads with SAMtools and minimap2 ( Li, 2018) and generates coverage tracks for regions of fixed size. In parallel, it queries the GoaT database ( Challis *et al.*, 2023) to identify all matching BUSCO lineages to run BUSCO ( Manni *et al.*, 2021). For the three domain-level BUSCO lineage, the pipeline aligns the BUSCO genes to the Uniprot Reference Proteomes database ( Bateman *et al.*, 2023) with DIAMOND ( Buchfink *et al.*, 2021) blastp. The genome is also split into chunks according to the density of the BUSCO genes from the closest taxonomically lineage, and each chunk is aligned to the Uniprot Reference Proteomes database with DIAMOND blastx. Genome sequences that have no hit are then chunked with seqtk and aligned to the NT database with blastn ( Altschul *et al.*, 1990). All those outputs are combined with the blobtools suite into a blobdir for visualisation.

All three pipelines were developed using the nf-core tooling ( Ewels *et al.*, 2020), use MultiQC ( Ewels *et al.*, 2016), and make extensive use of the Conda package manager, the Bioconda initiative ( Grüning *et al.*, 2018), the Biocontainers infrastructure ( da Veiga Leprevost *et al.*, 2017), and the Docker ( Merkel, 2014) and Singularity ( Kurtzer *et al.*, 2017) containerisation solutions.

Table 3 contains a list of relevant software tool versions and sources.

**Table 3.** Software tools: versions and sources.

Software tool	Version	Source
BEDTools	2.30.0	https://github.com/arq5x/bedtools2
Blast	2.14.0	ftp://ftp.ncbi.nlm.nih.gov/blast/executables/blast+/
BlobToolKit	4.3.7	https://github.com/blobtoolkit/blobtoolkit
BUSCO	5.4.3 and 5.5.0	https://gitlab.com/ezlab/busco
bwa-mem2	2.2.1	https://github.com/bwa-mem2/bwa-mem2
Cooler	0.8.11	https://github.com/open2c/cooler
DIAMOND	2.1.8	https://github.com/bbuchfink/diamond
fasta_windows	0.2.4	https://github.com/tolkit/fasta_windows
FastK	427104ea91c78c3b8b8b49f1a7d6bbeaa869ba1c	https://github.com/thegenemyers/FASTK
gEVAL	N/A	https://geval.org.uk/
GoaT CLI	0.2.5	https://github.com/genomehubs/goat-cli
Hifiasm	0.16.1-r375	https://github.com/chhylp123/hifiasm
HiGlass	44086069ee7d4d3f6f3f0012569789ec138f42b84aa44357826c0b6753eb28de	https://github.com/higlass/higlass
MerquryFK	d00d98157618f4e8d1a9190026b19b471055b22e	https://github.com/thegenemyers/MERQURY.FK
MitoHiFi	2	https://github.com/marcelauliano/MitoHiFi
MultiQC	1.14, 1.17, and 1.18	https://github.com/MultiQC/MultiQC
NCBI Datasets	15.12.0	https://github.com/ncbi/datasets
Nextflow	23.04.0-5857	https://github.com/nextflow-io/nextflow
PretextView	0.2	https://github.com/wtsi-hpag/PretextView
purge_dups	1.2.3	https://github.com/dfguan/purge_dups
samtools	1.16.1, 1.17, and 1.18	https://github.com/samtools/samtools
sanger-tol/genomenote	1.1.1	https://github.com/sanger-tol/genomenote
sanger-tol/readmapping	1.2.1	https://github.com/sanger-tol/readmapping
Seqtk	1.3	https://github.com/lh3/seqtk
Singularity	3.9.0	https://github.com/sylabs/singularity
YaHS	yahs-1.1.91eebc2	https://github.com/c-zhou/yahs

### Genome annotation

The BRAKER2 pipeline ( Brůna *et al.*, 2021) was used in the default protein mode to generate annotation for the *Nycteola revayana* assembly (GCA_947037095.2) in Ensembl Rapid Release at the EBI.

### Wellcome Sanger Institute – Legal and Governance

The materials that have contributed to this genome note have been supplied by a Darwin Tree of Life Partner. The submission of materials by a Darwin Tree of Life Partner is subject to the **‘Darwin Tree of Life Project Sampling Code of Practice’**, which can be found in full on the Darwin Tree of Life website here. By agreeing with and signing up to the Sampling Code of Practice, the Darwin Tree of Life Partner agrees they will meet the legal and ethical requirements and standards set out within this document in respect of all samples acquired for, and supplied to, the Darwin Tree of Life Project. 

Further, the Wellcome Sanger Institute employs a process whereby due diligence is carried out proportionate to the nature of the materials themselves, and the circumstances under which they have been/are to be collected and provided for use. The purpose of this is to address and mitigate any potential legal and/or ethical implications of receipt and use of the materials as part of the research project, and to ensure that in doing so we align with best practice wherever possible. The overarching areas of consideration are:

• Ethical review of provenance and sourcing of the material

• Legality of collection, transfer and use (national and international) 

Each transfer of samples is further undertaken according to a Research Collaboration Agreement or Material Transfer Agreement entered into by the Darwin Tree of Life Partner, Genome Research Limited (operating as the Wellcome Sanger Institute), and in some circumstances other Darwin Tree of Life collaborators.

## Data Availability

European Nucleotide Archive: *Nycteola revayana* (oak Nycteoline). Accession number PRJEB55450; https://identifiers.org/ena.embl/PRJEB55450 ( Wellcome Sanger Institute, 2023). The genome sequence is released openly for reuse. The *Nycteola revayana* genome sequencing initiative is part of the Darwin Tree of Life (DToL) project. All raw sequence data and the assembly have been deposited in INSDC databases. Raw data and assembly accession identifiers are reported in Table 1.

## Author information

Members of the University of Oxford and Wytham Woods Genome Acquisition Lab are listed here: https://doi.org/10.5281/zenodo.7125292.

Members of the Natural History Museum Genome Acquisition Lab are listed here: https://doi.org/10.5281/zenodo.7139035.

Members of the Darwin Tree of Life Barcoding collective are listed here: https://doi.org/10.5281/zenodo.4893703.

Members of the Wellcome Sanger Institute Tree of Life Management, Samples and Laboratory team are listed here: https://doi.org/10.5281/zenodo.10066175.

Members of Wellcome Sanger Institute Scientific Operations: Sequencing Operations are listed here: https://doi.org/10.5281/zenodo.10043364.

Members of the Wellcome Sanger Institute Tree of Life Core Informatics team are listed here: https://doi.org/10.5281/zenodo.10066637.

Members of the Tree of Life Core Informatics collective are listed here: https://doi.org/10.5281/zenodo.5013541.

Members of the Darwin Tree of Life Consortium are listed here: https://doi.org/10.5281/zenodo.4783558.
