# Supplementary material for: The genome sequence of the Oak Nycteoline moth, Nycteola revayana (Scopoli, 1772)
Source: Wellcome Open Res. 2024 May 15;9:258. [Version 1] doi: 10.12688/wellcomeopenres.21567.1 (PMC11391193; doi:10.12688/wellcomeopenres.21567.1)
Supplement: Peer review history

# Reviewer report: Reviewer response for version 1

**DOI:** 10.21956/wellcomeopenres.23839.r96547

**Published:** 2024-10-04

**Authors:** Pedro De G. Ribeiro

**Affiliations:** 1 Department of Ecology, Biology Centre of the Czech Academy of Sciences, České Budějovice, Czech Republic

Authors present a Data Note on the chromosome-level genome sequencing of the Oak Nycteoline moth ( *Nycteola revayana*) together with RNAseq. The Note is standard in terms of methods and results, following the quality of the Sanger Tree of Life/Darwin Tree of Life Projects, making it clear and useful for readers. 

 Authors provide a sufficient introduction and rationale behind sequencing this genome, and I would not consider that there is anything major (or minor) left behind.

Are sufficient details of methods and materials provided to allow replication by others?

Yes

Is the rationale for creating the dataset(s) clearly described?

Yes

Are the datasets clearly presented in a useable and accessible format?

Yes

Are the protocols appropriate and is the work technically sound?

Yes

Reviewer Expertise:

Evolutionary biology of butterflies including phylogenetics and biogeography.

I confirm that I have read this submission and believe that I have an appropriate level of expertise to confirm that it is of an acceptable scientific standard.

# Reviewer report: Reviewer response for version 1

**DOI:** 10.21956/wellcomeopenres.23839.r96549

**Published:** 2024-09-11

**Authors:** Alexander Kneubehl

**Affiliations:** 1 Department of Pediatrics, Baylor College of Medicine, Houston, TX, USA

Boyes *et al*. present a complete genome sequence of the Oak Nycteoline moth. This assembly was from a male specimen and generated 26 chromosomal pseudomolecules including the Z chromosome. The assembly was generated using PacBio HiFi sequencing with Illumina Hi-C. RNA sequencing was also performed for annotation purposes using BRAKER2.

**I only have two points that need further clarification:**

1. In the Background "... into adaptations to oak feeding" is this remarkable for this species? If so, more should be given as to why this is important.
2. In the Methods, what is "$HIC_TISSUE"?

Are sufficient details of methods and materials provided to allow replication by others?

Yes

Is the rationale for creating the dataset(s) clearly described?

Partly

Are the datasets clearly presented in a useable and accessible format?

Yes

Are the protocols appropriate and is the work technically sound?

Yes

Reviewer Expertise:

Bacterial genomics, arthropod genomics, viral genomics, vector-borne disease, molecular biology

I confirm that I have read this submission and believe that I have an appropriate level of expertise to confirm that it is of an acceptable scientific standard.

# Reviewer report: Reviewer response for version 1

**DOI:** 10.21956/wellcomeopenres.23839.r92998

**Published:** 2024-09-11

**Authors:** Rodolpho S.T Menezes

**Affiliations:** 1 Department of Biological Sciences, State University of Santa Cruz, Ilhéus, Brazil

The data note by Boyes *et al*. presents a genome assembly from an individual male of the moth *Nycteola revayana* (Scopoli, 1772). The final assembly is 621 Mb. This genome information will be interesting for comparative genomics investigation and also for evolution and conservation studies. The methodology is thorough and explained and the figures are well constructed, and the data note is well written.

 I have no notes to add.

Are sufficient details of methods and materials provided to allow replication by others?

Yes

Is the rationale for creating the dataset(s) clearly described?

Yes

Are the datasets clearly presented in a useable and accessible format?

Yes

Are the protocols appropriate and is the work technically sound?

Yes

Reviewer Expertise:

Cytogenetics, Phylogenomics, and Phylogeography.

I confirm that I have read this submission and believe that I have an appropriate level of expertise to confirm that it is of an acceptable scientific standard.
